# Intestinal mucosa-derived DNA methylation signatures in the penetrating intestinal mucosal lesions of Crohn’s disease

**DOI:** 10.1038/s41598-021-89087-6

**Published:** 2021-05-07

**Authors:** Yuan Li, Zhiming Wang, Xiuwen Wu, Gefei Wang, Guosheng Gu, Huajian Ren, Zhiwu Hong, Jianan Ren

**Affiliations:** 1 Research Institute of General Surgery, Jinling Hospital, Medical School of Nanjing University, Nanjing, 210002 China; 2 Department of General Surgery, The First Affiliated Hospital of Nanjing Medical University, Nanjing, 210029 China

**Keywords:** Clinical genetics, Epigenetics, Inflammatory bowel disease, Intestinal diseases

## Abstract

The purpose of this study was to evaluate genome-wide DNA methylation changes in intestinal mucosa tissue of adult patients with Crohn's disease comprehensively. DNA methylation chip was used to analyze abnormal methylation sites among penetrating and non-penetrating intestinal mucosa tissue of Crohn's disease and normal intestinal mucosa tissue of healthy controls. Methylation abnormalities of different locus were verified by pyrosequencing and quantitative polymerase chain reaction. Differential DNA methylation sites were participated in the positive regulation of apoptosis and the positive regulation of IL-8 production and were enriched in signaling pathways related to inflammatory bowel disease and extracellular matrix receptor interaction signaling pathways. Correlation analysis showed that the methylation abnormalities of HLA-DRB1 (r = − 0.62, P < 0.001), MUC1 (r = − 0.45, P = 0.01), YPEL5 (r = − 0.55, P = 0.001) and CBLB (r = − 0.62, P < 0.001) were significantly negatively correlated with their relative expression levels. The degree of methylation abnormality of MUC1 was negatively correlated with the disease activity score of Crohn's disease (r = − 0.50, P = 0.01). Apoptosis, interleukin-8 production and abnormal extracellular matrix might be involved in the mechanism of penetrating intestinal mucosal lesions in Crohn's disease. The degree of abnormal methylation of MUC1 was negatively correlated with the disease activity of Crohn's disease.

## Introduction

Crohn's disease (CD) was a chronic recurrent intestinal disease that primarily affected the end of the small intestine and the beginning of the colon^1^. Penetrating intestinal mucosal lesions^2^, including intestinal perforation and intestinal fistula, was one of the serious complications of CD. At present, the etiology of CD was still not clear, and the mainstream opinion was that CD was caused by the complex interaction of multiple factors (including genetic variation, intestinal flora, host immune system and environmental factors). Genome-wide association study (GWAS)^3^ had identified more than 100 CD-related susceptibilities genetic locus. However, in the actual study^4^, only a small proportion (13.6%) of CD patients were observed with genetic variation, suggesting that non-genetic factors (such as intestinal flora, environmental factors, etc.) accounted for a certain proportion of CD etiology. Epigenetics, as an important crucial connection between genetic factors and non-genetic factors^5^, was bound to play an important role in the pathogenesis of CD.

Early studies^6–9^ mainly focused on DNA methylation changes in peripheral blood of CD, while only a few studies^10,11^ noticed the changes in intestinal mucosal tissue of CD. The intestinal mucosal tissue was the most direct joint point among the human body, external environment and intestinal flora, and it contained a rich and complex intestinal immune system. Therefore, the DNA methylation status of the intestinal mucosal tissue of CD was worth further investigation. Besides, the research interests of previous studies^12,13^ on CD intestinal mucosal tissue methylation had focused on the secondary carcinogenesis of CD-related colitis, but were rarely involved in the more common penetrating intestinal mucosal lesions of CD. The mechanism of penetrating intestinal mucosal lesions in CD was still unknown. Once penetrating intestinal mucosal lesions occurred in CD patients, surgical intervention was often required, and medical risks and costs were greatly increased^14^. Therefore, exploring the pathogenesis of penetrating intestinal mucosal lesions in CD patients and screening molecular markers might be contributed to the early detection and intervention of the lesions.

The purpose of this study was to use DNA methylation chip to comprehensively evaluate the genome-wide DNA methylation changes in the intestinal mucosal tissue of adult CD patients, and to compare the DNA methylation status with that of healthy controls to screen the differential DNA methylation sites. By exploring different epigenetic regulation signaling pathways in the intestinal mucosal tissue of CD patients, new ideas could be provided to clarify the pathogenesis of CD and screen potential biomarkers, to improve the diagnosis and treatment of CD.

## Results

### Basic information of patients in both groups

All 7 CD patients enrolled in the CD group were male, with an average age of 31.3 ± 8.3 years. All sampling sites were ileum. After strict age and sex matching between CD group and healthy control group, gender and age data were consistent, and the sampling sites were all ileum. The baseline characteristics of the patients were shown in Supplementary Tables S1 and S2.

### Quality control of methylated chips

Principal component analysis (Supplementary Fig. S1) showed that the CD group (red and blue squares) and the control group (blue squares) were distributed in different areas in the two-dimensional space, indicating that the samples were reasonably grouped and had good in-group repeatability.

### Differential DNA methylation sites among CD penetrating and CD non-penetrating intestinal mucosal tissue and normal intestinal mucosal tissue

Comparisons of CD penetrating intestinal mucosal tissue with normal intestinal mucosal tissue, a total of 5200 different DNA methylation sites were screened. There were 2978 hypermethylation sites and 2222 demethylation sites. Comparison of CD penetrating intestinal mucosal tissue with non-penetrating intestinal mucosal tissue, a total of 3237 different DNA methylation sites were identified. There were 1157 hypermethylation sites and 2080 demethylation sites.

### Volcano plots

**Figure 1:**
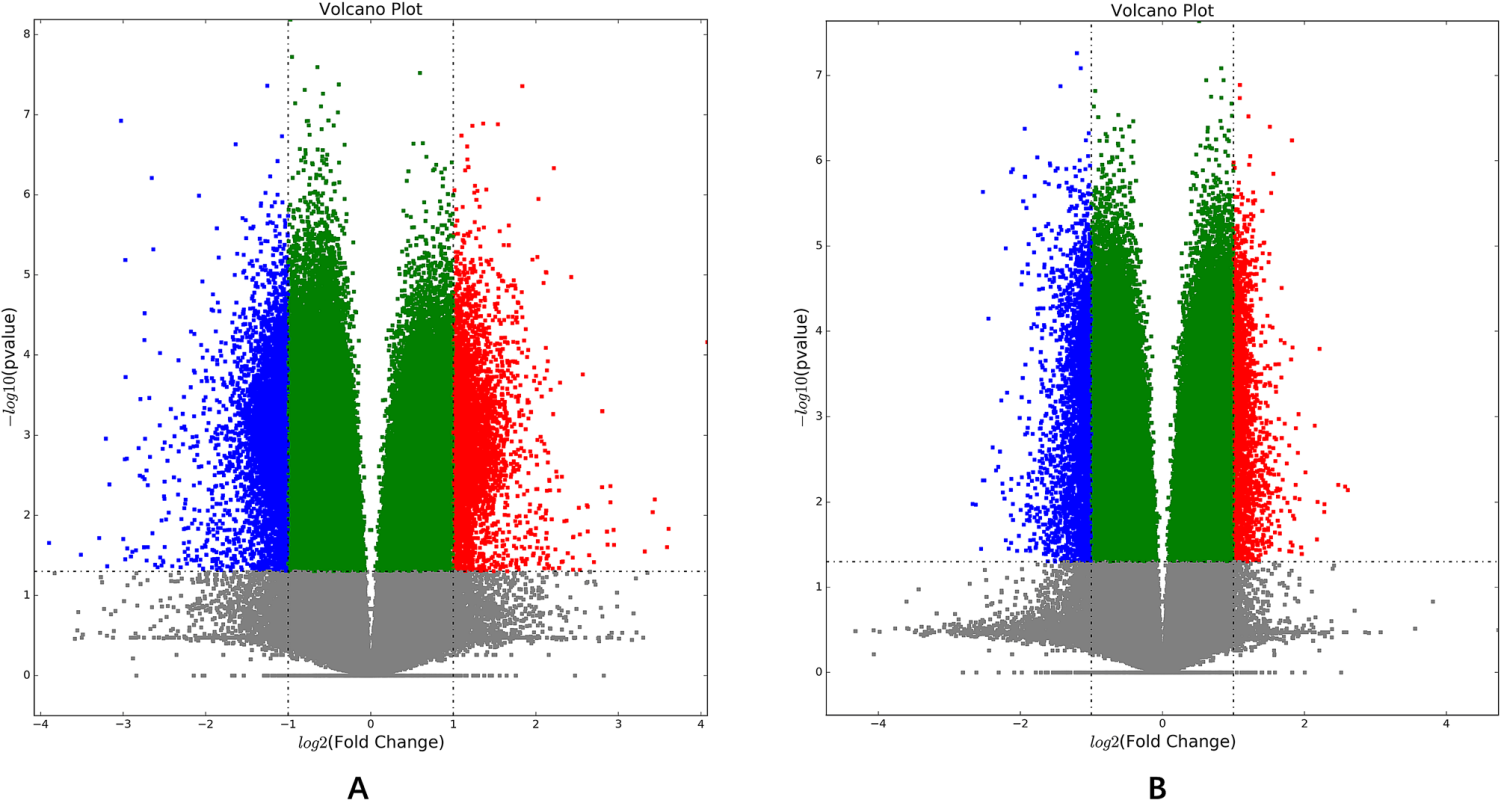
Volcano Plots. (**A**) Comparisons of differential DNA methylation sites between CD penetrating intestinal mucosal tissue with normal intestinal mucosal tissue; (**B**) Comparisons of differential DNA methylation sites between CD penetrating and non-penetrating intestinal mucosal tissue. Figures were performed with affy R package (Version 3.6.1, https://www.r-project.org).

Comparisons of CD penetrating intestinal mucosal tissue with normal intestinal mucosal tissue, several sites with the highest degree of hypermethylation and demethylation were screened by drawing volcano plots (Fig. 1A) as follows (Table 1): Hypermethylation sites (KCNJ13; GIGYF2, C7orf72 and HLA-DRB1); Demethylation sites (HERPUD2, MUC1, and TMTC2). Comparison of CD penetrating intestinal mucosal tissue with non-penetrating intestinal mucosal tissue, several sites with the highest degree of hypermethylation and demethylation were screened by drawing volcano plots (Fig. 1B) as follows (Table 2): Hypermethylation sites (MTSS1, YPEL5, EFCAB11 and CBLB); Demethylation sites (PLEKHG1, LINC01506 and KIAA0753).

**Table 1:** Differential DNA methylation sites information between CD penetrating intestinal mucosal tissue with normal intestinal mucosal tissue.

Methylation state	Gene name	Target ID	Gene ID	Delta_Beta value	Diffscore value	Chromosome
Up	KCNJ13;GIGYF2	cg03946744	3769;26058	0.2422366	25.99213	2
Up	C7orf72	cg20233834	100130988	0.1600762	68.88832	7
Up	HLA-DRB1	cg09949906	3123	0.2346621	16.65833	6
Down	HERPUD2	cg23759826	64224	− 0.09881615	− 73.61388	7
Down	MUC1	cg00930306	4582	− 0.2432761	− 62.10949	1
Down	TMTC2	cg07690222	160335	− 0.1874332	− 64.20343	12

**Table 2:** Differential DNA methylation sites information between CD penetrating intestinal mucosal tissue with non-penetrating intestinal mucosal tissue.

Methylation state	Gene name	Target ID	Gene ID	Delta_ Beta value	Diffscore value	Chromosome
Up	MTSS1	cg13992976	9788	0.1865852	48.12816	8
Up	YPEL5	cg26462319	51646	0.1361087	67.35131	2
Up	EFCAB11	cg26886948	90141	0.2068419	65.19701	14
Up	CBLB	cg21116912	868	0.1975454	63.97843	3
Down	PLEKHG1	cg26116556	57480	− 0.1961553	− 70.83961	6
Down	LINC01506	cg10940369	101927015	− 0.16296	− 63.23583	9

### Cluster analysis

#### Comparisons of CD penetrating intestinal mucosal tissue with normal intestinal mucosal tissue

**Figure 2:**
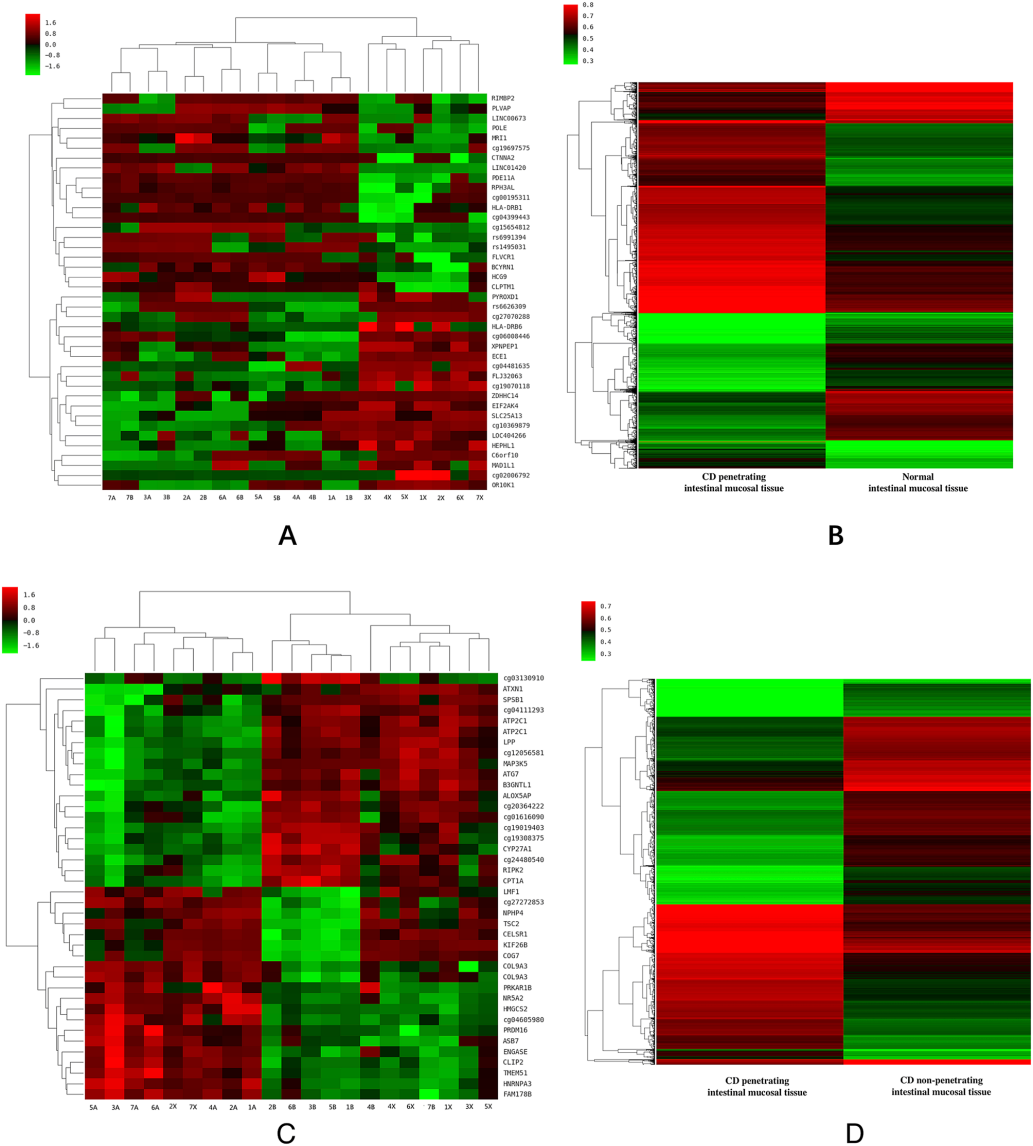
Cluster analysis of CD penetrating intestinal mucosal tissue and normal intestinal mucosal tissue. (**A**) The overall; (**B**) Top 20 highest degree of hypermethylation and demethylation sites; Cluster analysis of CD penetrating and non-penetrating intestinal mucosal tissue. (**C**) The overall; (**D**) Top 20 highest degree of hypermethylation and demethylation sites. The serial numbers at the bottom of (**A**) and (**C**) presented: (**A**) CD penetrating intestinal mucosal tissue; (**B**) CD non-penetrating intestinal mucosal tissue; X: normal intestinal mucosal tissue. Figures were performed with affy R package (Version 3.6.1, https://www.r-project.org).

According to the 5200 differential DNA methylation sites screened by the methylation chips, the CD penetrating intestinal mucosal tissue were compared with the normal intestinal mucosal tissue for cluster analysis. We found that the methylation status of the two groups were significantly different (Fig. 2A).

According to the clustering analysis of previously screened top 20 highest degree of hypermethylation and demethylation sites (Fig. 2B), CD intestinal mucosal tissue and normal intestinal mucosal tissue could be distinguished and classified completely.

#### Comparisons of CD penetrating intestinal mucosal tissue with non-penetrating intestinal mucosal tissue

According to the 3237 differential DNA methylation sites screened by the methylation chips, CD penetrating intestinal mucosal tissue were compared with non-penetrating intestinal mucosal tissue for cluster analysis. It was found that the methylation status of the two groups were significantly different (Fig. 2C).

According to the clustering analysis of previously screened top 20 highest degree of hypermethylation and demethylation sites (Fig. 2D), CD penetrating and non-penetrating intestinal mucosal tissue and normal mucosal tissue could be distinguished and classified completely.

### GO analysis

#### Comparisons of CD penetrating intestinal mucosal tissue with normal intestinal mucosal tissue

**Figure 3:**
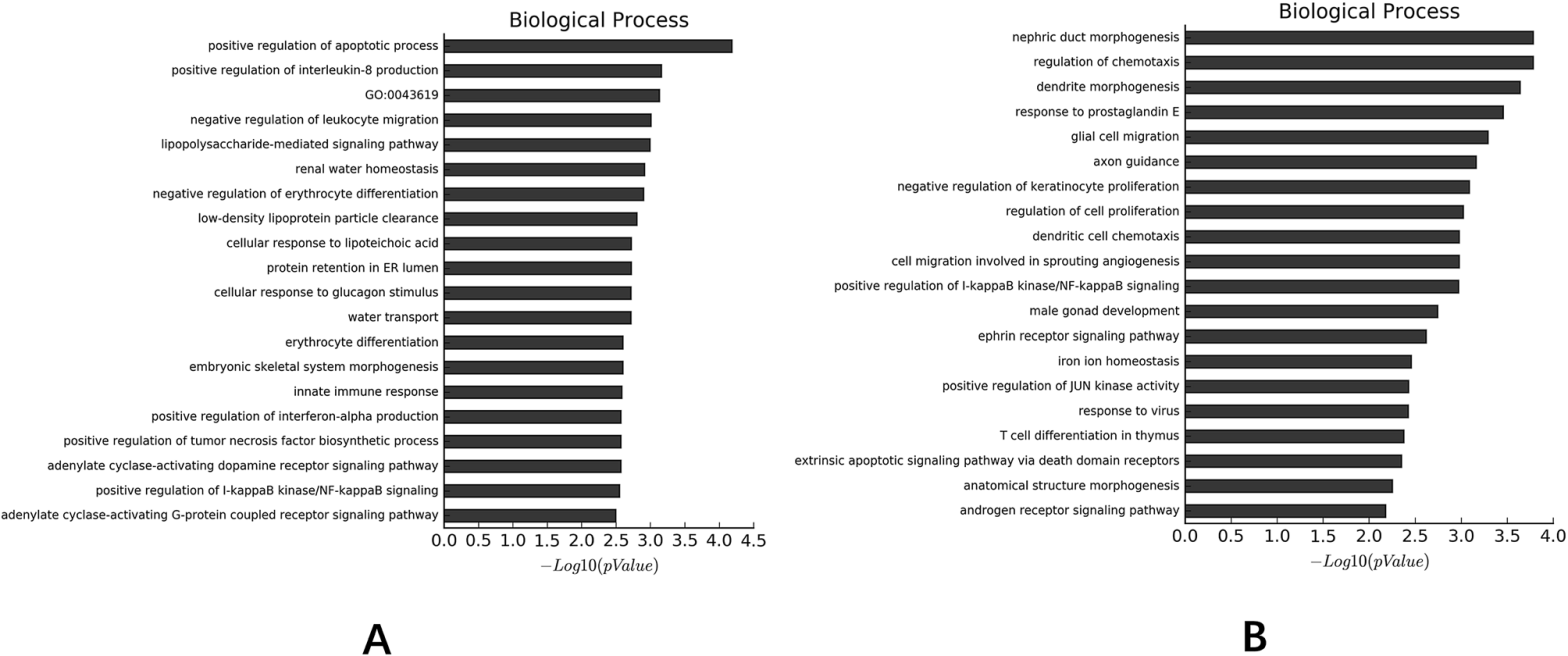
Go analysis. (**A**) Comparisons of differential DNA methylation sites between CD penetrating intestinal mucosal tissue with normal intestinal mucosal tissue; (**B**) Comparisons of differential DNA methylation sites between CD penetrating and non-penetrating intestinal mucosal tissue. GO data were downloaded from GO website (http://geneontology.org).

As shown in Fig. 3A, differential DNA methylation sites were enriched in the positive regulation of the apoptotic process and the positive regulation of interleukin-8 production in the biological process.

#### Comparisons of CD penetrating intestinal mucosal tissue with non-penetrating intestinal mucosal tissue

As shown in Fig. 3B, differential DNA methylation sites were enriched in the renal duct development and the regulation of chemotaxis in the biological process.

### KEGG analysis

#### Comparisons of CD penetrating intestinal mucosal tissue with normal intestinal mucosal tissue

**Figure 4:**
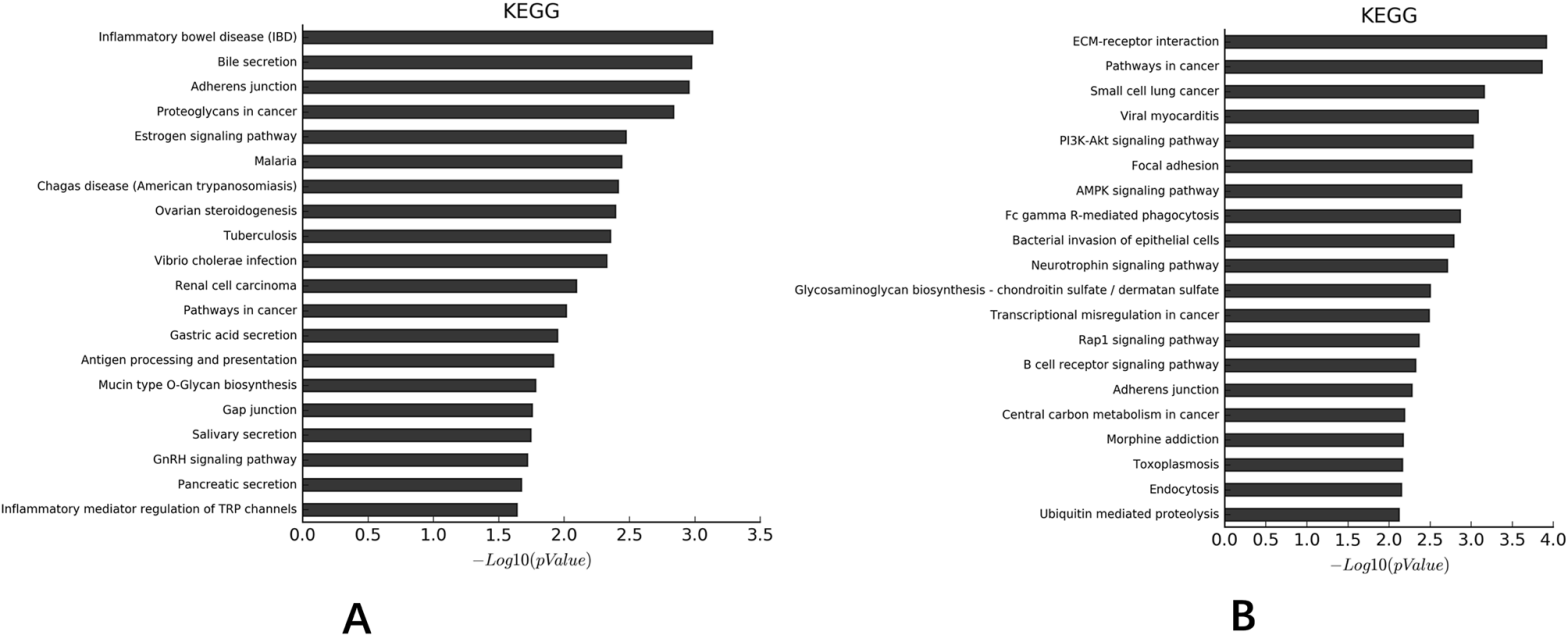
KEGG analysis. (**A**) Comparisons of differential DNA methylation sites between CD penetrating intestinal mucosal tissue with normal intestinal mucosal tissue; (**B**) Comparisons of differential DNA methylation sites between CD penetrating and non-penetrating intestinal mucosal tissue. KEGG pathways were downloaded from KEGG website (https://www.kegg.jp). The permission was provided by Kanehisa laboratory.

Pathway analysis of differential DNA methylation sites with KEGG database showed that the differentially expressed sites were mainly concentrated in signal pathways associated with IBD (Fig. 4A).

#### Comparisons of CD penetrating intestinal mucosal tissue with non-penetrating intestinal mucosal tissue

Pathway analysis of differential DNA methylation sites with KEGG database showed that the differentially expressed sites were mainly concentrated in signal pathways associated with ECM-receptor interaction signal pathway (Fig. 4B).

### Verification of methylation chip results

#### The verification of pyrosequencing

**Figure 5:**
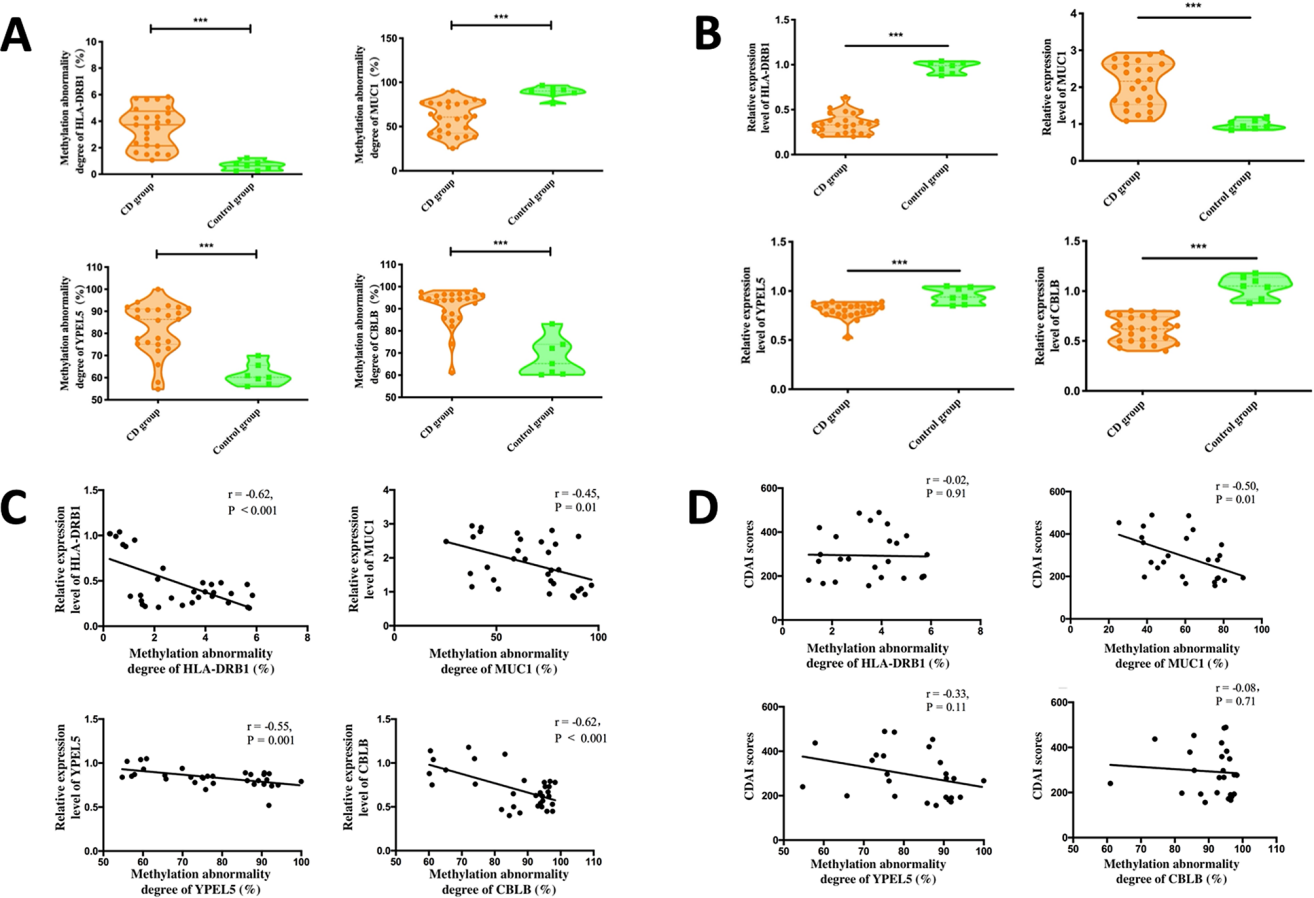
(**A**) The methylation abnormality degrees of HLA-DRB1, YPEL5, CBLB and MUC1 in the intestinal mucosal tissue of the CD group were verified by pyrosequencing. (**B**) The relative expression levels of HLA-DRB1, YPEL5, CBLB and MUC1in the intestinal mucosal tissue of CD group were tested by qPCR. (**C**) Correlation between methylation abnormality degrees of HLA-DRB1, YPEL5, CBLB and MUC1 and gene expression level. (**D**) Correlation between methylation degree of MUC1 and disease activity of CD. Figures were performed with Prism (Version 6.0c, https://www.graphpad.com/scientific-software/prism/).

As shown in Fig. 5A, the methylation abnormality degrees of HLA-DRB1, YPEL5 and CBLB in the intestinal mucosal tissue of the CD group were significantly higher than that of the control group (P < 0.001, as shown in figure ***, the same below), while that of MUC1 was significantly lower (P < 0.001).

#### The verification of gene expression levels

As shown in Fig. 5B, compared with the control group, the relative expression levels of HLA-DRB1, YPEL5 and CBLB in the intestinal mucosal tissue of CD group were significantly reduced by qPCR (P < 0.001), while the relative expression levels of MUC1 were significantly increased (P < 0.001).

#### Correlation between methylation abnormality degrees and gene expression levels

According to the correlation analysis (Fig. 5C), the methylation abnormality degrees of HLA-DRB1 (r = − 0.62, P < 0.001), MUC1 (r = − 0.45, P = 0.01), YPEL5 (r = − 0.55, P = 0.001) and CBLB (r = − 0.62, P < 0.001) were found to have significant negative correlations with their relative expression levels in the intestinal mucosal tissue in CD group and control group.

#### Correlation between methylation degree of MUC1 and disease activity of CD

The CDAI scores of CD group were evaluated and calculated on the day of sampling. Through correlation analysis (Fig. 5D), methylation abnormality degree of MUC1 in the intestinal mucosa of CD group was negatively correlated with the corresponding CDAI scores (r = 0.50, P = 0.01), while methylation abnormality degrees of HLA-DRB1 (r = 0.02, P = 0.91), YPEL5 (r = 0.33, P = 0.11) and CBLB (r = 0.08, P = 0.71) were not correlated with CDAI scores.

#### The fold changes of DEGs among GSE95095, GSE83448, GSE103027 and GSE102133

**Figure 6:**
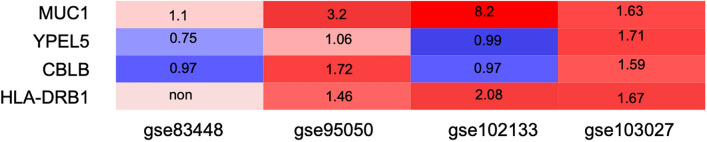
The fold changes of DEGs among GSE95095, GSE83448, GSE103027 and GSE102133. The GEO databases were obtained from NCBI-GEO databases (https://www.ncbi.nlm.nih.gov/geo). Figures were performed with affy R package (Version 3.6.1, https://www.r-project.org).

Heat maps were generated to visualize the fold changes (FC) of expressed genes between CD and normal controls from different studies. As shown in Fig. 6, heat maps were generated with R based on the FC of selected DEGs (HLA-DRB1, YPEL5, CBLB and MUC1) in GSE95095, GSE83448, GSE103027 and GSE102133. The red columns represented up-regulated (FC > 1), while the blue ones represented downregulated (FC > 1). The depth of color was positively correlated with FC values.

## Discussion

In this study, we used Illumina HD 850 K DNA methylation chips were used to screen the abnormal DNA methylation sites in intestinal mucosal tissue of CD with penetrating intestinal mucosal lesions in the study. We recruited a large cohort of CD patients and health control, and developed the pyrosequencing was used to verify the differential sites of DNA methylation screened by methylated chips, and MUC1, a new molecular marker. We obtained potential early clinical diagnosis of CD-penetrating intestinal mucosal lesions by this novel assay.

DNA methylation played an important role in gene expression^15,16^. In most cases, gene promoter methyl attachment (hypermethylation) was associated with gene silencing or inactivation. Conversely, hypomethylation of gene promoters activated transcription. Therefore, hypermethylation of DNA sites resulted in down-regulated expression of corresponding genes, while demethylation resulted in up-regulated expression of the corresponding genes^17^.

By strictly matching the age and gender of the experimental group and the control group in the early stage, the bias of age and gender on the final results was minimized. A total of 5200 sites of differential DNA methylation were screened by comparing CD penetrating intestinal mucosal tissue with normal intestinal mucosal tissue. There were 2978 hypermethylation sites and 2222 demethylation sites. The highest degree of differential hypermethylation sites included KCNJ13; GIGYF2, C7orf72, and HLA-DRB1, and differential demethylation sites included HERPUD2, MUC1, and TMTC2. A total of 3237 differential DNA methylation sites were identified by comparing CD penetrating intestinal mucosal tissue with non-penetrating intestinal mucosal tissue. There were 1157 hypermethylation sites and 2080 demethylation sites. The most differential hypermethylation locus included MTSS1, YPEL5, EFCAB11 and CBLB, and differential demethylation locus included PLEKHG1, LINC01506 and KIAA0753. Most of the differential hypermethylated and hypomethylated CpG sites were located in the body. Only few of them, like PLEKHG1 and KIAA0753 were located in 5′UTR. The differentiated methylated regions directly affected gene expression. This was worth in-depth study in the future.

By screening the differential methylation sites, CD penetrating intestinal mucosal tissue, non-penetrating intestinal mucosal tissue and normal intestinal mucosal tissue could be well distinguished. The results also showed that the differential DNA methylation sites were involved in the positive regulatory process of apoptosis and the positive regulatory process of IL-8 production, and were enriched in the inflammatory bowel disease related signaling pathways and extracellular matrix receptor interaction signaling pathways.

The methylation degrees of HLA-DRB1, MUC1, YPEL5 and CBLB were verified by pyrosequencing method. Compared with the control group, the methylation levels of HLA-DRB1, YPEL5 and CBLB sites in the intestinal mucosal tissue of the CD group were significantly increased, while the methylation level of MUC1 site was significantly reduced. Comparing the relative expression levels of the four genes, it was found that the relative expression levels of HLA-DRB1, YPEL5 and CBLB in the CD group were significantly reduced, while the relative expression level of MUC1 gene was significantly increased. Correlation analysis showed that the abnormal degrees of the four methylation sites was negatively correlated with their relative expression levels. The analysis of methylation levels and CD disease activity showed that the abnormal methylated status of MUC1 was negatively correlated with CDAI scores.

In comparing the differential DNA methylation sites screened by CD penetrating intestinal mucosal tissue and normal intestinal mucosal tissue, C7orf72^18^ and HLA-DRB1^19^ were the susceptibility genetic locus that had been confirmed by GWAS studies to be related to the pathogenesis of CD. Using GWAS, HLA-DRB1 was selected as the susceptibility gene of CD in CD patients in Chinese^20^, Japanese^21^, British^22^ and African American^23^ populations. Studies had also found that HLA-DRB1 was related to the polymorphism of IL-10 gene^24^, and IL-10 gene abnormality was involved in the important mechanisms of CD^25^.

MUC1, as a selective methylation site, was involved in the regulation of mucin-1 expression. Mucin-1 was a major product which secreted by goblet cells^26^ and was a component of ECM^27^. Recent studies^28–32^ had found that the expression of MUC1 in CD was significantly increased. This was consistent with the fact that methylation of MUC1 was reduced in this study. Interestingly, the MUC1 expression in CD was reduced in earlier studies^33,34^. In addition, studies^35–38^ had confirmed that MUC1 was confirmed to be involved in multiple apoptosis signaling pathways.

In comparing the differential DNA methylation sites screened by CD penetrating intestinal mucosal tissue and non-penetrating intestinal mucosal tissue, YPEL5^39^ had been proved to be involved in the mitochondrial dependent apoptosis process caused by DNA damage. CBLB was regulated by the NF-κb signaling pathway and was involved in the activation of T cells and macrophages^40^.

Through screening of top 20 highest degree of hypermethylation and demethylation sites, CD penetrating intestinal mucosal tissue, non-penetrating intestinal mucosal tissue and normal intestinal mucosal tissue could be well distinguished, indicating that these differential methylation sites could be used as molecular markers to guide the diagnosis and therapy of CD in the future.

By GO analysis^41,42^, we found that the differential DNA methylation sites were involved in the positive regulatory process of apoptosis and the positive regulatory process of IL-8 production. It was found that the apoptosis process was significantly enhanced in CD^43^, especially in the penetrating lesions. Tight junction proteins were also shown to be closely related to the apoptosis process in CD^44^. The secretion of IL-8 in CD was also significantly increased^45,46^, and some studies^47^ had used it as a biomarker. IL-8 and MUC1 were both important factors involved in the pathophysiological process of CD^28,38^.

Through KEGG analysis^48,49^, our study found that the methylation site of CD was enriched in the classic IBD pathway. Previous research^50^ had explored that the epigenetic modifications of the classic pathway in IBD (nuclear factor Kappa-B Signaling Pathway) and its impact on IBD. The results confirmed again that the selected differential methylation sites could be used as molecular markers for the diagnosis of CD. The methylation sites associated with penetrating inflammatory lesions were concentrated in the related pathways of ECM receptor interaction. Recent studies^51,52^ had also found that ECM-receptor interaction was closely related to the course of CD.As mentioned earlier, mucin-1 was one of the components of ECM. The formation of CD penetrating intestinal mucosal lesions was suggested to be the result of the destruction of ECM^53^, while the abnormal MUC1 expression product mucin-1 just explained the origin of CD penetrating intestinal mucosal lesions, indicating that intestinal mucosal penetrating lesions are correlated with ECM. However, as mentioned above, the expression of MUC1 in CD was still controversial and needs further study to clarify.

In this study, high-throughput technology was used to screen the differential DNA methylation sites related to CD penetrating intestinal mucosal diseases through methylation chips. Using the selected methylation sites, CD penetrating intestinal mucosal tissue and non-penetrating intestinal mucosal tissue could be well distinguished from normal intestinal mucosal tissue. Among the selected methylation sites, C7orf72^18^ and HLA-DRB1^19^ had been confirmed to be related to CD by GWAS. As mentioned previously, MUC1^35–38^ and YPEL5^39^ were genes involved in the process of apoptosis, which proved that CD penetrating intestinal mucosal lesions were related to apoptosis. Methylation abnormalities in MUC1 also suggested that CD penetrating intestinal mucosal lesions were associated with ECM abnormalities. The results of GO analysis and KEGG analysis confirmed the above conclusions again.

Pyrosequencing^54^ was a common method for quantifying the abnormal degrees of methylation. Using pyrosequencing method, it was found that the abnormal degree of four methylation sites in CD group was consistent with the results of the methylation chip in the previous study. QPCR was used to detect the relative expression levels of the four genes, which was also consistent with the abnormal degrees of methylation. Correlation analysis showed that the abnormal degrees of methylation and relative expression levels of the four sites were significantly correlated, which confirmed that the four differential methylation sites screened by methylated chips, namely HLA-DRB1, MUC1, YPEL5 and CBLB, were reliable and correlated with the process of CD penetrating intestinal mucosal lesions.

CDAI scores of CD group was further analyzed, and correlation analysis was used to explore the relationship between methylation abnormality degree and disease activity degree. It was found that methylation abnormality degree of MUC1 was negatively correlated with CDAI score. The results suggested that MUC1 could be used as a molecular marker of CD activity. In the future, the disease activity of CD could be predicted by detecting the methylation level of MUC1. For CD patients with atypical clinical manifestations and insignificant endoscopic or imaging manifestations, the discovery of novel biomarkers might be helped to make a definite diagnosis earlier, providing a possibility for early intervention of CD disease course.

We had also compared the four significant DEGs (HLA-DRB1, YPEL5, CBLB and MUC1) among four independent studies. By comparing the DEGs, some trends of gene expressions were consistent with our study, especially the MUC1. Among the four independent studies, the expression levels of MUC1 were all up-regulated , which were the same as our study.

In previous studies on DNA methylation of CD, peripheral blood samples^7,8,11,55–57^ were often used. Some DNA hypermethylated genes in peripheral blood of CD were considered as promising new biomarkers for CD^58–60^. However, intestinal mucosal tissue might be more suitable for epigenetic studies as a bridge between the internal environment of the body and the external environment and intestinal flora. Furthermore, research^61^ that related to DNA methylation in intestinal mucosal tissue as biomarkers had achieved some initial results. Although existing research^62^ confirmed a moderate-strong correlation between methylation levels in colon biopsies and peripheral blood samples. However, the methylation levels in peripheral blood samples as biomarkers remained to be debated^63^. The results pointed to a new research direction. The differences of methylation profiles between intestinal mucosal tissue and peripheral blood specimens could be conducted in the further study. In the field of inflammatory bowel diseases, previous studies^64–66^ had focused on the relationship between DNA methylation abnormalities and CD-related colorectal canceration.

The sample size of this study was small. Although the selected differential methylation sites suggested the possible mechanism of CD penetrating intestinal mucosal lesions, further studies were needed to verify the above findings. As mentioned above, the expression of MUC1 gene was different in different literatures, which also need further research and verification. Secondly, only four different methylation sites that had been studied a lot in the past were selected for verification. The verification of other sites need to be completed in the future. Finally, as a participant in the process of apoptosis and the formation of ECM, the mechanism of MUC1 in CD penetrating intestinal mucosal lesions also need further study.

In summary, through methylation chip technology, this study screened out gene locus related to the pathogenesis of CD penetrating intestinal mucosal diseases, such as C7orf72, HLA-DRB1, MUC1, YPEL5, CBLB, etc. CD—penetrating intestinal mucosal lesions were also associated with apoptosis, IL-8 production, and ECM abnormalities. The methylation of HLA-DRB1, YPEL5 and CBLB was abnormally increased in CD penetrating intestinal mucosal lesions, while the methylation of MUC1 was decreased. The methylation abnormality of MUC1 was negatively correlated with CD disease activity.

At present, CD was a disease with unclear pathogenesis. With the increasing incidence of CD all over the world, more and more patients were troubled by its diverse clinical manifestations and complicated complications. The study provided a new direction for the study of the pathogenesis of CD. By exploring the abnormal sites of DNA methylation in CD, it could provide provide an effective basis for screening novel biomarkers and therapeutic targets, so as to improve the prognosis of patients and reduce medical expenses..

## Methods

### Ethical approval

The study was conducted in accordance with the *Declaration of Helsinki* and was approved by the Ethics Committee of Jinling Hospital. The study was registered on the Clinical Trials (No. : NCT03272152). Informed consent was obtained from all patients and healthy volunteers before their enrollment in this study.

### Patients

The study included two cohorts of patients.

1. Seven CD patients who underwent colonoscopy in Jinling Hospital in November 2016 were randomly selected among outpatients. All 7 patients were diagnosed with CD and were active. 7 healthy people were matched with age and sex as the control group.
2. Twenty-five CD patients and seven healthy controls who underwent colonoscopy in Jinling Hospital in January 2017 were randomly selected for colonoscopy in Jinling Hospital. All 25 patients were diagnosed with CD, were active, and had penetrating intestinal mucosal lesions.

Inclusion criteria:

1. Age: > 18 years old;
2. A definitive diagnosis of CD was based on the results of multiple examinations, such as colonoscopy, enteroscopy, gastroscope, computed tomography, enterography, histopathological examination, and blood tests (including routine blood examination, erythrocyte sedimentation rate, C‐reactive protein, and autoimmune‐related antibodies);
3. Imaging and endoscopic examination confirmed penetrating intestinal mucosal lesions (perforation or fistula);
4. Crohn's disease activity index (CDAI) is greater than 150;
5. At least two following laboratory indexes of blood sedimentation to meet: erythrocyte sedimentation rate > 30 mm/h, hemoglobin < 12 g/dl (male) or < 11.5 g/d (female), platelet > 350*10^9^/L, or C—reactive protein more than 2 times higher than normal;
6. No smoking history or quit smoking for more than 6 months;
7. Participate in clinical trials voluntarily and sign informed consent.

Exclusion criteria:

1. The lesion was located in the upper digestive tract;
2. Existing other diseases, such as systemic diseases, liver and kidney dysfunction, malignant tumors, lung diseases, etc.;
3. Treatment history of immunosuppressive agents and biological agents within nearly half one year;
4. Pregnancy or lactation;
5. Patients who participated in other clinical studies within the last 3 months;
6. Patients who are not suitable for electronic colonoscopy;
7. Patients with other conditions that the investigator considers inappropriate for participation.

### Data collection

All patient data were collected from the electronic medical record system of Jinling Hospital. Data include age, gender, CDAI score, blood indexes, etc.

### Grouping and acquisition of intestinal mucosa samples

All enrolled individuals underwent electronic colonoscopy and intestinal mucosal tissue was taken from the ileum or ileum side of the ileocolon anastomotic site. Among them, intestinal mucosa tissues were taken from the penetrating lesion site as well as the normal site in the CD penetrating group and the CD non-penetrating group. Healthy volunteers in the control group also underwent colonoscopy and had their intestinal mucosa collected.

All the intestinal mucosa samples were immediately frozen in liquid nitrogen, and then frozen at − 80℃ for later use.

### DNA extraction and bisulphite treatment

DNA was extracted from tissue samples using a QIAamp DNA Mini Kit (*QiagenTM*, Valencia, California, USA). DNA purity was assessed by measuring the A260/A280 ratio using a NanoDrop (*Thermo Scientific*) and DNA quality was checked by agarose gel electrophoresis for a strong band at high molecular weight. The concentration, purity and integrity of DNA were tested in accordance with the requirements of Ilumina 850 K BeadChips according to the Illumina methylation protocol. Bisulphite treatment of each sample was undertaken using the Zymo EZ DNA Methylation kit(*ZYMO Research*, Orange County, California, USA). BeadChips were processed with robotics and analyzed using the Illumina Hi-Scan system.

### Illumina Human Methylation 850K microarray

Genomestudio software (Version 2011.1, Illumina, Inc., Albany, New York, USA) was used to standardize the processing of chip scan data, calculate standardized signal value and evaluate the situation of detected genes (detection standard: P < 0.05). Illumina Human Methylation 850K microarray profiling and data analysis were performed by *Oebiotech* (Shanghai, China).

### Validation by pyrosequencing

Pyrosequencing assays were performed to validate the results obtained from previous findings of HLA-DRB1(cg24760581), MUC1(cg00930306), YPEL5(cg26462319), CBLB(cg21116912). PyroMark Assay Design software (Version 2.0, Qiagen, Valencia, California, USA) was used to Design PCR primers and detect mutated sites. Primer sequence is shown in Table 3.

**Table 3:** Pyrosequencing primer.

Pyrosequencing primer	Primer sequences		Fragment length (nt)
HLA-DRB1	F	GAGTAAAGGAGATGGAGGGAATAT	24
	R	AAAACATCCACAAAATCACATTTTCTAAT	29
MUC1	F	TGGAGGGGAGGTGGAGTTT	19
	R	ACCCCCCCCCCAACCCAC	18
YPEL5	F	GGTATTTTGGTGTTGTTGTTAAATATAGT	29
	R	TAACACCCCCCAAATAAATACTAAC	25
CBLB	F	TTGTTTTGTTTGGGTGGTAAAAAAT	25
	R	CTAAACTCCTTCTACAATCCTACTC	25

*Note* F represented the forward primer of 5′-3′, and R represented the reverse primer of 3′-5'.

### Quantitative Real-time PCR

The primers were synthesized by Invitrogen Biotechnology Co., LTD, China, and the sequences were shown in Table 4.

**Table 4:** Quantitative Real-time PCR primers.

Quantitative Real-time PCR primer		Primer sequence	Primer length (bp)
β-actin	Forward	5′-CAGGGCGTGATGGTGGGCA-3'	253
	REVERSE	5′-CAAACATCATCTGGGTCATCTTCTC-3'	
HLA-DRB1	Forward	5′-TCCCTGAGTGAGACTTGCCTG-3'	209
	Reverse	5′-CTCCGTCCCATTGAAGAAATG-3'	
MUC1	Forward	5′-CAGCCACTTCTGCCAACTTG-3'	124
	Reverse	5′-AGCTCACCAGCCCAAACAG-3'	
YPEL5	Forward	5′-AGCACCAGAGCCCATTCTTC-3'	125
	Reverse	5′-CAACCCAGCAGTTCTGTCCC-3'	
CBLB	Forward	5′-AGTGCTTATGCGGAAACACAG-3′	147
	Reverse	5′-TTTATGCTAGGGAGGAGGGTG-3′	

### Statistics

Statistical analysis and Figures were performed with Prism (GraphPad Software, Inc., version 6.0c). Categorical variables, such as mortality, were analyzed by Fisher’s exact test. Continuous variables are shown as the mean ± S.D. Mann–Whitney U test was used to compare continuous variables between groups^67^. Intergroup comparisons of continuous variables were performed using the Mann–Whitney U test, and intergroup comparisons of classified variables were performed using the chi-square test or Fisher's exact test^67^. The correlation between methylation degree and relative gene expression and the correlation between methylation degree and CDAI score were both analyzed by Pearson correlation analysis. *P*-values < 0.001 was regarded as statistically different and indicated by *** in figures. All *P*-values were two-sided.

### Data source

The original datasets comparing the gene expression profiles between CD and normal controls were downloaded from NCBI GEO databases^68^. The accession number was GSE95095, GSE83448, GSE103027 and GSE102133 respectively. The microarray data of GSE95095 was based on GPL14951 (Illumina HumanHT-12 WG-DASL V4.0 R2 expression beadchip). The microarray data of GSE83448 was bsaed on GPL18134 (CodeLink Human Whole Genome Array). The microarray data of GSE103027 was bsaed on GPL13534 (Illumina HumanMethylation450 beadChip). and the microarray data of GSE102133 was bsaed on GPL6244 (Affymetrix Human Gene 1.0 ST Array).

### Data pre-processing and differential expression analysis

Robust multi-array average (RMA) approach was performed for background correction and normalization^69^. Batch effects were removed in all data. The original GEO data were then converted into expression measures using affy R package (R version 3.6.1)^70^. Limma R package was subsequently employed for identifying differentially expressed genes (DEGs). P < 0.05 and absolute log2FC > 1 were chosen as the cut-off criteria based on Benjamini & Hochberg (BH) procedure. Intersect function in R was used for identifying the common DEGs among GSE95095, GSE83448, GSE103027 and GSE102133.

## Supplementary Information

Supplementary Figure S1.

Supplementary Table S1.

Supplementary Table S2.

Supplementary Table S3.

Supplementary Table S4.

## Data Availability

No additional data are available.
