# Supplementary figures and images for: Intestinal mucosa-derived DNA methylation signatures in the penetrating intestinal mucosal lesions of Crohn’s disease
Source: Sci Rep. 2021 May 7;11:9771. doi: 10.1038/s41598-021-89087-6 (PMC8105344; doi:10.1038/s41598-021-89087-6)

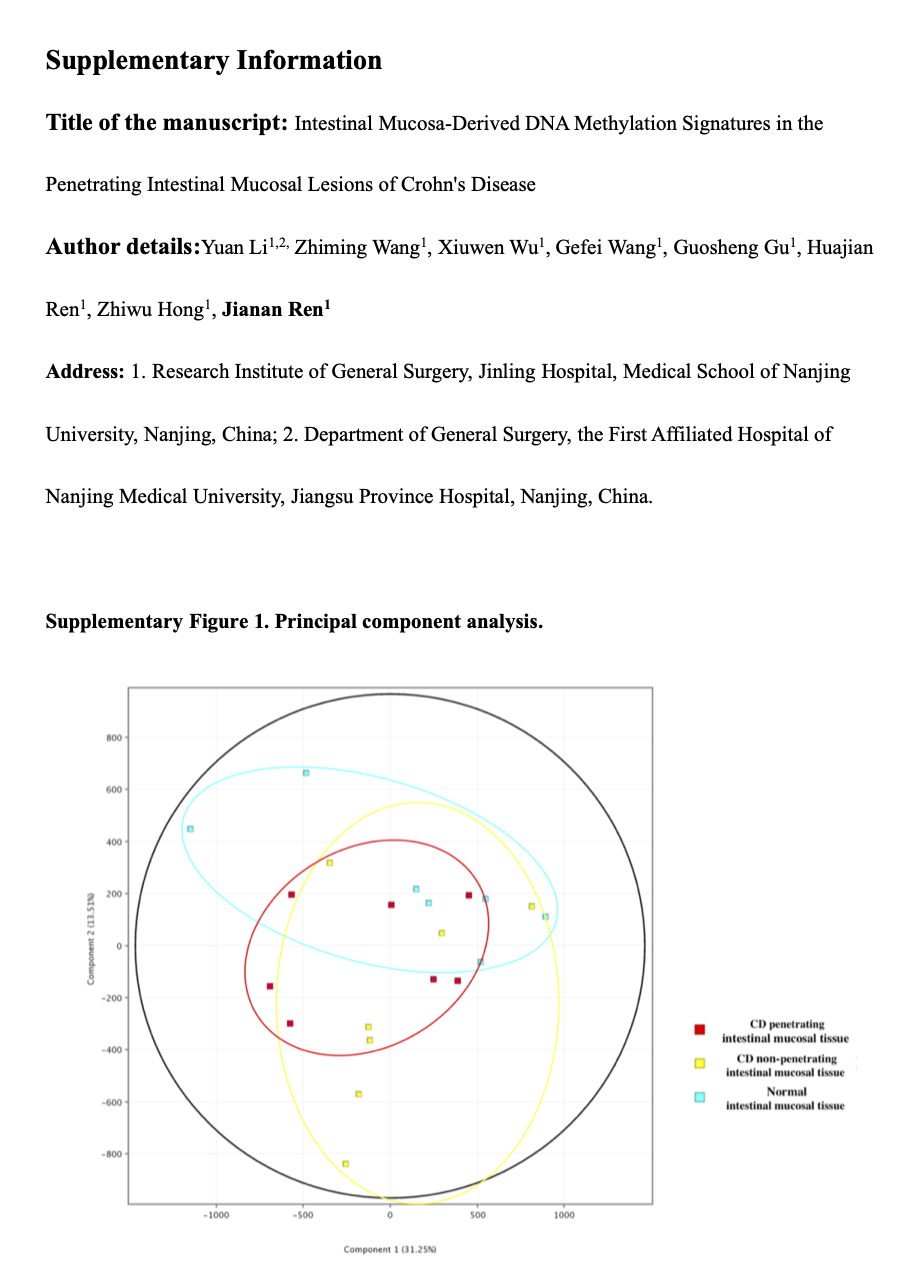

Supplement: Supplementary file 1 — Supplementary Figure S1. [file 41598_2021_89087_MOESM1_ESM.tiff]
