# Supplementary material for: Intestinal mucosa-derived DNA methylation signatures in the penetrating intestinal mucosal lesions of Crohn’s disease
Source: Sci Rep. 2021 May 7;11:9771. doi: 10.1038/s41598-021-89087-6 (PMC8105344; doi:10.1038/s41598-021-89087-6)
Supplement: Supplementary file 2 — Supplementary Table S1. [file 41598_2021_89087_MOESM2_ESM.docx]

**Title of the manuscript:** Intestinal Mucosa-Derived DNA Methylation Signatures in the Penetrating Intestinal Mucosal Lesions of Crohn's Disease
**Author details:** Yuan Li1,2, Zhiming Wang1, Xiuwen Wu1, Gefei Wang1, Guosheng Gu1, Huajian Ren1, Zhiwu Hong1, **Jianan Ren1
Address:** 1. Research Institute of General Surgery, Jinling Hospital, Medical School of Nanjing University, Nanjing, China; 2. Department of General Surgery, the First Affiliated Hospital of Nanjing Medical University, Jiangsu Province Hospital, Nanjing, China.

**Supplementary table 1.** The baseline characteristics of patients in the first cohort

| No. of patients | Sex | Age | Age at diagnosis：A | Disease location：L | Disease behavior：B |
| --- | --- | --- | --- | --- | --- |
| 1 | Male | 33 | A2 | L1 | B3+P |
| 2 | Male | 45 | A3 | L3 | B3 |
| 3 | Male | 27 | A2 | L1 | B3+P |
| 4 | Male | 39 | A2 | L1 | B3 |
| 5 | Male | 26 | A2 | L1 | B3 |
| 6 | Male | 28 | A2 | L1 | B3 |
| 7 | Male | 21 | A2 | L1 | B3 |
