# Supplementary material for: Intestinal mucosa-derived DNA methylation signatures in the penetrating intestinal mucosal lesions of Crohn’s disease
Source: Sci Rep. 2021 May 7;11:9771. doi: 10.1038/s41598-021-89087-6 (PMC8105344; doi:10.1038/s41598-021-89087-6)
Supplement: Supplementary file 3 — Supplementary Table S2. [file 41598_2021_89087_MOESM3_ESM.docx]

**Title of the manuscript:** Intestinal Mucosa-Derived DNA Methylation Signatures in the Penetrating Intestinal Mucosal Lesions of Crohn's Disease
**Author details:** Yuan Li1,2, Zhiming Wang1, Xiuwen Wu1, Gefei Wang1, Guosheng Gu1, Huajian Ren1, Zhiwu Hong1, **Jianan Ren1
Address:** 1. Research Institute of General Surgery, Jinling Hospital, Medical School of Nanjing University, Nanjing, China; 2. Department of General Surgery, the First Affiliated Hospital of Nanjing Medical University, Jiangsu Province Hospital, Nanjing, China.

**Supplementary table 2.** The baseline characteristics of patients in the second cohort

|  | CD  （n=25） | Control  （n=7） | P value |
| --- | --- | --- | --- |
| Sex（Male, %） | 17（68.0%） | 5（71.4%） | 1.000 |
| Age（Years） | 30.5±8.0 | 26.7±5.4 | 0.255 |
