# Supplementary material for: Intestinal mucosa-derived DNA methylation signatures in the penetrating intestinal mucosal lesions of Crohn’s disease
Source: Sci Rep. 2021 May 7;11:9771. doi: 10.1038/s41598-021-89087-6 (PMC8105344; doi:10.1038/s41598-021-89087-6)
Supplement: Supplementary file 4 — Supplementary Table S3. [file 41598_2021_89087_MOESM4_ESM.docx]

**Title of the manuscript:** Intestinal Mucosa-Derived DNA Methylation Signatures in the Penetrating Intestinal Mucosal Lesions of Crohn's Disease
**Author details:** Yuan Li1,2, Zhiming Wang1, Xiuwen Wu1, Gefei Wang1, Guosheng Gu1, Huajian Ren1, Zhiwu Hong1, **Jianan Ren1
Address:** 1. Research Institute of General Surgery, Jinling Hospital, Medical School of Nanjing University, Nanjing, China; 2. Department of General Surgery, the First Affiliated Hospital of Nanjing Medical University, Jiangsu Province Hospital, Nanjing, China.

**Supplementary table 3.** The methylation status of differential DNA methylation sites according to the comparisons of CD penetrating intestinal mucosal tissue with normal intestinal mucosal tissue

| Methy-lation status | Gene Name | Target ID | Gene ID | Delta_Beta | Diffscore | Methy-lation regions | CHR |
| --- | --- | --- | --- | --- | --- | --- | --- |
| up | KCNJ13;GIGYF2 | cg03946744 | 3769;26058 | 0.2422366 | 25.99213 | TSS1500;Body | 2 |
| up | C7orf72 | cg20233834 | 100130988 | 0.1600762 | 68.88832 | Body | 7 |
| up | HLA-DRB1 | cg09949906 | 3123 | 0.2346621 | 16.65833 | Body | 6 |
| down | HERPUD2 | cg23759826 | 64224 | -0.09881615 | -73.61388 | Body | 7 |
| down | MUC1 | cg00930306 | 4582 | -0.2432761 | -62.10949 | Body | 1 |
| down | TMTC2 | cg07690222 | 160335 | -0.1874332 | -64.20343 | Body | 12 |
