# Supplementary material for: Intestinal mucosa-derived DNA methylation signatures in the penetrating intestinal mucosal lesions of Crohn’s disease
Source: Sci Rep. 2021 May 7;11:9771. doi: 10.1038/s41598-021-89087-6 (PMC8105344; doi:10.1038/s41598-021-89087-6)
Supplement: Supplementary file 5 — Supplementary Table S4. [file 41598_2021_89087_MOESM5_ESM.docx]

**Title of the manuscript:** Intestinal Mucosa-Derived DNA Methylation Signatures in the Penetrating Intestinal Mucosal Lesions of Crohn's Disease
**Author details:** Yuan Li1,2, Zhiming Wang1, Xiuwen Wu1, Gefei Wang1, Guosheng Gu1, Huajian Ren1, Zhiwu Hong1, **Jianan Ren1
Address:** 1. Research Institute of General Surgery, Jinling Hospital, Medical School of Nanjing University, Nanjing, China; 2. Department of General Surgery, the First Affiliated Hospital of Nanjing Medical University, Jiangsu Province Hospital, Nanjing, China.

**Supplementary table 4.** The methylation status of differential DNA methylation sites according to the comparisons of CD penetrating intestinal mucosal tissue with non-penetrating intestinal mucosal tissue

| Methy-lation status | Gene Name | Target ID | Gene ID | Delta_Beta | Diffscore | Methy-lation regions | CHR |
| --- | --- | --- | --- | --- | --- | --- | --- |
| up | MTSS1 | cg13992976 | 9788 | 0.1865852 | 48.12816 | Body | 8 |
| up | YPEL5 | cg26462319 | 51646 | 0.1361087 | 67.35131 | Body | 2 |
| up | EFCAB11 | cg26886948 | 90141 | 0.2068419 | 65.19701 | Body | 14 |
| up | CBLB | cg21116912 | 868 | 0.1975454 | 63.97843 | Body | 3 |
| down | PLEKHG1 | cg26116556 | 57480 | -0.1961553 | -70.83961 | 5'UTR | 6 |
| down | LINC01506 | cg10940369 | 101927015 | -0.16296 | -63.23583 | TSS1500 | 9 |
| down | KIAA0753 | cg21696827 | 9851 | -0.164979 | -60.54529 | 5'UTR | 17 |
